# Multiple biomarkers predict disease severity, progression and mortality in COPD

**DOI:** 10.1186/s12931-017-0597-7

**Published:** 2017-06-13

**Authors:** Rachel L. Zemans, Sean Jacobson, Jason Keene, Katerina Kechris, Bruce E. Miller, Ruth Tal-Singer, Russell P. Bowler

**Affiliations:** 1 Division of Pulmonary, Critical Care, and Sleep Medicine, Department of Medicine, National Jewish Health, 1400 Jackson St., Denver, CO 80206 USA; 2 Division of Pulmonary Sciences and Critical Care Medicine, Department of Medicine, University of Colorado Denver, University of Colorado Anschutz Medical Campus, Research Building 2, 9th Floor, 12700 E. 19th Ave., Aurora, CO USA; 3 Department of Biostatistics and Informatics, University of Colorado Denver, Colorado School of Public Health, Mail Stop B119, 13001 E. 17th Place, Aurora, CO 80045 USA; 4 GlaxoSmithKline R&D, 709 Swedeland Road #1539, King Of Prussia, PA USA

**Keywords:** COPD, Biomarker, Cohort study

## Abstract

**Background:**

Chronic obstructive pulmonary disease (COPD) is a heterogeneous disease characterized by multiple subtypes and variable disease progression. Blood biomarkers have been variably associated with subtype, severity, and disease progression. Just as combined clinical variables are more highly predictive of outcomes than individual clinical variables, we hypothesized that multiple biomarkers may be more informative than individual biomarkers to predict subtypes, disease severity, disease progression, and mortality.

**Methods:**

Fibrinogen, C-Reactive Protein (CRP), surfactant protein D (SP-D), soluble Receptor for Advanced Glycation Endproducts (sRAGE), and Club Cell Secretory Protein (CC16) were measured in the plasma of 1465 subjects from the COPDGene cohort and 2746 subjects from the ECLIPSE cohort. Regression analysis was performed to determine whether these biomarkers, individually or in combination, were predictive of subtypes, disease severity, disease progression, or mortality, after adjustment for clinical covariates.

**Results:**

In COPDGene, the best combinations of biomarkers were: CC16, sRAGE, fibrinogen, CRP, and SP-D for airflow limitation (*p* < 10^−4^), SP-D, CRP, sRAGE and fibrinogen for emphysema (*p* < 10^−3^), CC16, fibrinogen, and sRAGE for decline in FEV_1_ (*p* < 0.05) and progression of emphysema (*p* < 10^−3^), and all five biomarkers together for mortality (*p* < 0.05). All associations except mortality were validated in ECLIPSE. The combination of SP-D, CRP, and fibrinogen was the best model for mortality in ECLIPSE (*p* < 0.05), and this combination was also significant in COPDGene.

**Conclusion:**

This comprehensive analysis of two large cohorts revealed that combinations of biomarkers improve predictive value compared with clinical variables and individual biomarkers for relevant cross-sectional and longitudinal COPD outcomes.

**Electronic supplementary material:**

The online version of this article (doi:10.1186/s12931-017-0597-7) contains supplementary material, which is available to authorized users.

## Background

Chronic obstructive pulmonary disease (COPD) is a global health burden that affects 10% of the world’s population and results in 3 million deaths and $44 billion in health care costs annually. Aside from smoking cessation and oxygen, no available therapies prolong survival or prevent disease progression, with few promising novel drugs in the pipeline [1]. One reason for this is the heterogeneity and complexity of the disease. COPD has multiple subtypes, including emphysema and the frequent exacerbator subtype [2, 3]. In addition, disease progression and mortality are variable and difficult to predict [4, 5]. Although clinical variables such as age, smoking history, dyspnea, exacerbation history, and body mass index (BMI) are somewhat useful to model these subtypes, assess disease severity, and predict disease progression, [6, 7] a large amount of unexplained variance remains.

Since the heterogeneity of COPD extends to the molecular level, there is growing interest in biomarkers to assess disease heterogeneity and predict progression. Biomarkers might identify subgroups of patients who would benefit from specific interventions or may serve as surrogate endpoints, thus enhancing statistical power and reducing the cost of clinical trials. Ultimately, biomarkers may facilitate prognosis and allow us to cater therapies to individual patents (i.e., precision medicine). Moreover, detection of subclinical disease through biomarkers could lead to interventions (e.g., smoking cessation) that could prevent the development of overt COPD. Finally, the identification of biomarkers associated with COPD subtypes or severity may stimulate basic research into the mechanisms underlying the pathogenesis of COPD and identify novel therapeutic targets.

Previous studies have identified several blood protein biomarkers of varying value in predicting COPD outcomes (Additional file 1: Table S1) [2]. Fibrinogen and CRP, markers of inflammation, may correlate with disease severity and risk of exacerbations [3, 4, 8–15]. sRAGE, which dampens inflammation, is inversely correlated with emphysema and airflow limitation [5, 16]. These observations have cemented our understanding that COPD is an inflammatory disease [10]. In fact, both fibrinogen and sRAGE have been considered by the U.S. Food and Drug Administration (FDA) and the European Medicines Agency (EMA) for approval as biomarkers for COPD. Proteins that derive from lung parenchymal cells have also been associated with COPD: SP-D and CC16 with airflow limitation [4, 17, 18] and SP-D with emphysema [5]. However, previous biomarker studies have several limitations. Most have focused on the relationship between biomarkers and cross-sectional outcomes such as subtype and disease severity, information that can be obtained by routine clinical testing. Perhaps the greatest clinical utility of biomarkers lies in their ability to predict disease progression, which is highly variable among COPD patients [4, 5]. The role of biomarkers in predicting longitudinal outcomes has been addressed in a limited number of studies. Fibrinogen and CRP tend to be elevated in patients with frequent exacerbations, but the extent to which biomarkers can predict *future* exacerbations is unclear [3, 10, 12, 14, 17, 19]. Decline in FEV_1_ is accelerated but highly variable amongst COPD patients; [4, 20, 21] some evidence suggests that CC16 [4, 22] and sRAGE [23] may be predictive. sRAGE and SP-D have been linked to progression of emphysema [5]. CRP, fibrinogen, CC16, and SP-D have been shown to be associated with mortality, although there are conflicting reports [2, 9–12, 15, 24, 25]. Another limitation of previous biomarker studies is that most examined a single biomarker. Just as combined clinical variables are more highly predictive of outcomes than individual clinical variables, [6, 26] we hypothesized that multiple biomarkers may be more powerful than individual biomarkers. Some precedent exists for the use of multiple biomarkers in COPD [10, 11, 14] and other diseases [27]. Finally, most COPD biomarker studies examined only one cohort, [5, 10, 11, 14] sometimes a small, single-site cohort, raising the possibility that findings may not be broadly applicable.

As most biomarker studies have been limited to assessing the relationship between individual biomarkers and cross-sectional outcomes and have been performed on a single cohort of patients, we aimed to determine whether a panel of a several biomarkers combined, as measured in two large, independent cohorts, would be more strongly predictive of important disease outcomes, particularly longitudinal outcomes, than individual biomarkers and clinical variables alone. Based on the literature, we evaluated the efficacy of five biomarkers - sRAGE, SP-D, fibrinogen, CC16, and CRP - both individually and in combination, at predicting airflow limitation, severity of emphysema, exacerbations, decline in FEV_1_, progression of emphysema, and mortality in the COPDGene and ECLIPSE cohorts.

## Methods

### Study design

Details of the COPDGene and ECLIPSE study protocols, including recruitment, data collection, and longitudinal follow-up are described in the online supplement (Additional File 3) and previous publications [28, 29]. COPDGene (NCT02445183) enrolled 10,300 subjects ages 45–80, of which plasma was collected from 1465 subjects. ECLIPSE (NCT00292552) enrolled 2746 subjects with complete data including biomarkers. Spirometry and high resolution CT scans were performed, and sRAGE, SP-D, high sensitivity (hs) CRP, fibrinogen, and CC16 levels were measured [10, 16–18].

### Clinical subtypes

COPD was defined by post-bronchodilator forced expiratory volume in the first second (FEV_1_) to forced vital capacity (FVC) ratio of <0.70. Smoker controls were current or former smokers without evidence of airflow limitation (FEV_1_/FVC ≥ 0.70). Emphysema was defined by the percent of voxels with Hounsfield Units (HU) < −950 (%LAA) on CT. Severity of emphysema was classified as none (LAA < 5%), mild (LAA 5–10%), moderate (LAA 10–20%), or severe (LAA > 20%) [30, 31]. Air trapping was measured by 3D Slicer. Air trapping was defined by the percent of voxels with HU < −856 on expiratory images. Airway wall thickness at an internal perimeter of 10 mm (pi10) was calculated as described previously [32]. Subjects were classified as having chronic bronchitis if they reported cough productive of sputum present daily for at least 3 months per year, at least 2 years in a row. Longitudinal follow-up (LFU) interviews by telephone or internet were conducted every six months. The number of exacerbations per year was determined. Moderate exacerbations were defined as those treated with steroids and/or antibiotics; severe exacerbations were defined as those resulting in hospitalization. Decline in FEV_1_ (ml/year) was calculated. Progression of emphysema was calculated as change in %LAA per year. All-cause mortality was determined.

### Statistical analysis

Because of non-normality, biomarker values were log transformed. Additional file 1: Table S2 lists statistical models and covariates, which were selected based on previous literature [3–7, 10, 11]. R (v 3.2.0) was used. Akaike Information Criteria (AIC) was used to determine how well a model fit. R^2^ for clinical covariates (no biomarkers) is reported; the R^2^ reported for biomarker(s) refers to the R^2^ of the biomarkers(s) over clinical covariates alone. p-values were determined by two-sided t-tests (or z-tests for the beta, negative binomial and logistic regression, and Cox proportional hazards) for the null hypothesis that β coefficients for biomarker-outcome associations were zero. Biomarker(s) were considered to improve the model if the AIC was lower than clinical covariates alone and p ≤ 0.05. The best combination of biomarkers for a given outcome in the COPDGene cohort was considered to be validated by ECLIPSE if the same combination of biomarkers statistically significantly improved the AIC over clinical covariates alone.

## Results

### Demographics

Baseline characteristics of the COPDGene and ECLIPSE cohorts are shown in Additional file 1: Tables S3 and S4.

All analyses performed on the COPDGene and ECLIPSE cohorts are shown in Additional file 2: Figure S5 and S6, respectively, with the best model in each cohort highlighted in yellow. The best model in COPDGene is shown in red font on the ECLIPSE analysis (Additional file 1: Table S6).

### Airflow limitation (FEV_1_/FVC and FEV_1_)

**Fig. 1:**
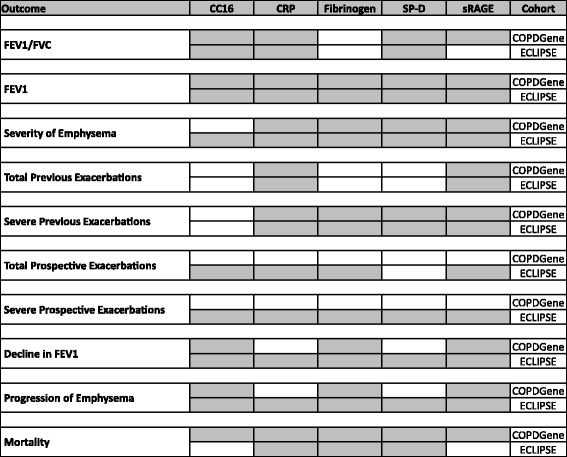
Best Models. The combinations of biomarkers that constituted the best models for each outcome in each cohort are shaded

In the COPDGene cohort, CC16, sRAGE, and CRP were each individually associated with FEV_1_/FVC after adjustment for clinical covariates (Additional file 1: Tables S7 and S5). However, the best model (lowest AIC) in the COPDGene cohort was the combination of CC16, SP-D, CRP, and sRAGE (additional R^2^ = 0.086 over clinical covariates), and this combination also statistically significantly improved the model in ECLIPSE (Additional file 1: Tables S7 and S6). In both cohorts, every individual biomarker was significantly associated with FEV_1_, but the combination of all five biomarkers was the most highly associated (Table 1, Fig. 1, Additional file 2: Figure S1, Additional file 1: Tables S5 and S6).

**Table 1:** Biomarkers associated with FEV_1_

	COPDGene			ECLIPSE		
Biomarker(s)	β	R^2^	AIC	β	R^2^	AIC
CC16	13.85*	0.02	13,467*	19.77*	0.02	25,641*
SP-D	−12.03*	0.01	13,453*	−20.12*	0.03	25,635*
sRAGE	16.43*	0.02	13,332*	48.43*	0.10	22,421*
CRP	−12.27*	0.06	13,404*	−16.92*	0.09	25,205*
Fibrinogen	−47.40*	0.04	13,188*	−85.39*	0.11	23,109*
CC16, Fibrinogen, sRAGE, CRP, SP-D		0.13	12,951*^a^		0.24	19,148*^a^

Analysis by linear regression. Race was the only covariate. The R^2^ reported for biomarkers refers to the R^2^ of the biomarkers(s) over clinical covariates alone. *p < 10^-5^ in a two-sided t-test for the null hypothesis that β = 0.

^a^Best model

### Emphysema

In the COPDGene cohort, SP-D and sRAGE were each individually associated with emphysema after adjusting for clinical covariates (Table 2, Additional file 1: Table S5, Additional file 2: Figure S2). The best model was SP-D, sRAGE, CRP, and fibrinogen combined (Table 2 and Fig. 1). Both the role of SP-D and sRAGE individually and the combination of SP-D, sRAGE, CRP, and fibrinogen were validated in ECLIPSE (Table 2, Additional file 1: Table S6 and Fig. 1).

**Table 2:** Biomarkers associated with severity of emphysema

	COPDGene			ECLIPSE		
Biomarker(s)	β	Pseudo R^2^ CU	AIC	β	Pseudo R^2^ CU	AIC
None		0.41	1608		0.53	4747
CC16	−0.35	−0.0007	1609	0.26	0.05	4610
CRP	0.28	0.002	1607	−0.02	0.04	4588
SP-D	−0.696	0.004	1604*	−1.02	0.04	4583*
Fibrinogen	1.07	0.02	1582	0.16	0.13	4276
sRAGE	−1.79	0.02	1567*	−2.67	0.18	4019*
SP-D, sRAGE, CRP, Fibrinogen		0.04	1550*^a^		0.30	3470*

Analysis performed by ordinal logistic regression. CU: Cragg & Uhler’s. Covariates were FEV_1_, age, smoking status, gender, race, CT scanner, and BMI. The R^2^ reported for clinical covariates (no biomarkers) is given. The R^2^ reported for biomarkers refers to the R^2^ of the biomarkers(s) over clinical covariates alone. *p < 10^−3^ in a two-sided t-test for the null hypothesis that β = 0

^a^Best model

### Exacerbations

In both cohorts, the combination of sRAGE and CRP best modeled *total* exacerbation frequency over the previous 12 months (Additional file 1: Tables S5, S6 and S8A), whereas SP-D, CRP, sRAGE, and fibrinogen together best modeled previous *severe* exacerbations (Additional file 1: Tables S5, S6 and S8B, Fig. 1). In the COPDGene cohort, no biomarker(s) was significantly predictive of *future* total or severe exacerbations after adjustment for prior exacerbations and other clinical covariates (Additional file 1: Tables S5 and S9, Fig. 1).

### Decline in FEV_1_

In COPDGene, fibrinogen predicted decline in FEV_1_; the best model was CC16, sRAGE, and fibrinogen (Table 3, Additional file 1: Table S5, Fig. 1). In ECLIPSE, these findings were validated but the combination of all five biomarkers was most highly predictive of decline in FEV_1_ (Table 3, Additional file 1: Table S6, Fig. 1).

**Table 3:** Biomarkers associated with decline in FEV1

	COPDGene			ECLIPSE		
Biomarker(s)	β	R^2^m	AIC	β	R^2^m	AIC
None		0.42	1348		0.34	5611
CC16	−0.003	0.03	1318	0.020	0.03	5358*
SP-D	0.014	0.000003	1358	−0.013	0.08	5289
sRAGE	0.001	0.02	1315	0.016	0.04	4607
CRP	0.006	0.03	1330	0.008	0.00	5400*
Fibrinogen	0.046	0.03	1303*	0.022	0.02	5067
CC16, Fibrinogen, sRAGE		0.06	1268*^a^		0.07	4026*

Analysis performed by linear mixed model. Covariates were age, time, gender, height, smoking status, pack years, age^2^, height^2^. The R^2^ reported for clinical covariates (no biomarkers) is given. The R^2^ reported for biomarkers refers to the R^2^ of the biomarkers(s) over clinical covariates alone. *p < 0.05 in a two-sided t-test for the null hypothesis that β = 0

^a^Best model

### Progression of emphysema

After controlling for BMI, female gender, and ongoing cigarette smoking, factors which have previously been identified as risk factors for decline in CT density, [5] the combination of CC16, fibrinogen, and sRAGE was most highly predictive in the COPDGene cohort. This combination was validated in the ECLIPSE cohort, but the combination of all five biomarkers together was more highly predictive (Table 4, Additional file 1: Tables S5 and S6, Fig. 1).

**Table 4:** Biomarkers associated with progression of emphysema

	COPDGene			ECLIPSE		
Biomarker(s)	β	R^2^m	AIC	β	R^2^m	AIC
None		0.357	7281		0.49	27,783
SP-D	0.045	0.002	7271	0.1722	0.008	27,042
CRP	0.0023	0.002	7264	−0.0004	−0.006	26,891
CC16	−0.419	0.002	7256*	−0.16	0.005	27,056
sRAGE	−0.169	0.005	7103	−0.765	0.04	24,804*
Fibrinogen	−0.218	−0.004	7066	−0.867	0.006	24,968*
CC16, Fibrinogen, sRAGE		0.005	7010*^a^		0.043	21,866*

Analysis performed by linear mixed model. Covariates were FEV_1_, age, time, smoking status, gender, CT scanner, and BMI. The R^2^ reported for clinical covariates (no biomarkers) is given. The R^2^ reported for biomarkers refers to the R^2^ of the biomarkers(s) over clinical covariates alone. *p < 10^−3^ in a two-sided t-test for the null hypothesis that β = 0

^a^Best model

### Mortality

BMI, airflow limitation, dyspnea, and exercise capacity (BODE), are moderately predictive of mortality in COPD [6, 7]. To determine whether additional clinical variables improve the model, we performed a stepwise Cox Proportional Hazards analysis with BODE and other variables known to be associated with mortality. The best model in the COPDGene cohort was BODE + age^2^ + age + gender + exacerbation history, and this was validated in ECLIPSE (Table 5, Additional file 1: Tables S5 and S6).

**Table 5:** Clinical variables associated with mortality

	COPDGene		ECLIPSE	
Clinical Variable	AIC	R^2^	AIC	R^2^
BODE	1604*	0.41	3099*	0.20
BODE + Age^2^ + Age	1597*	0.45	3068*	0.29
BODE + Gender	1580*	0.50	3099*	0.21
BODE + Severe Exacerbations	1598*	0.44	3091*	0.23
BODE + Gender + Severe Exacerbations	1575*	0.53	3091*	0.23
BODE + Age^2^ + Age + Severe Exacerbations	1590*	0.48	3061*^a^	0.31
BODE + Age^2^ + Age + Gender	1575*	0.53	3069*	0.29
BODE + Age^2^ + Age + Gender + Severe Exacerbations	1568*^a^	0.56	3062*	0.31

Analysis performed by Cox Proportional Hazards. The R^2^ reported for clinical covariates (no biomarkers) is given. The R^2^ reported for biomarkers refers to the R^2^ of the biomarkers(s) over clinical covariates alone. *p < 10^−13^ in a two-sided t-test for the null hypothesis that β = 0

^a^Best model

In the COPDGene cohort, CC16 and SP-D were each individually predictive of mortality, and all five biomarkers was the best model (Table 6, Fig. 1). The combination of all five biomarkers was not validated in ECLIPSE (Table 6, Additional file 1: Table S6); however, the best model in ECLIPSE – the combination of CRP, fibrinogen, and SP-D – was also significant in COPDGene. Of note, when analyzed by C-statistic, none of the biomarkers were associated with mortality in either cohort (Additional file 1: Table S13).

**Table 6:** Biomarkers associated with mortality

	COPDGene			ECLIPSE		
Biomarker(s)	β	R^2^	AIC	β	R^2^	AIC
None		0.56	1568		0.31	3062
CC16	−0.91	0.01	1565*	0.06	0.02	2874
SP-D	0.96	0.02	1565*	0.79	0.03	2868*
sRAGE	0.43	0.00	1555	0.45	−0.03	1754
CRP	0.21	0.00	1569	0.36	0.01	2900*
Fibrinogen	−0.59	0.00	1528	1.61	0.02	2502*
CRP, Fibrinogen, SP-D		0.04	1523*		0.06	2249*^a^
CC16, CRP, Fibrinogen, SP-D, sRAGE		0.04	1509*^a^		−0.02	1276

Analysis performed by Cox Proportional Hazards. Covariates were BODE, age^2^, gender, and severe exacerbations. The R^2^ reported for clinical covariates (no biomarkers) is given. The R^2^ reported for biomarkers refers to the R^2^ of the biomarkers(s) over clinical covariates alone. *p < 0.02 in a two-sided t-test for the null hypothesis that β = 0

^a^Best model

## Discussion

COPD is a complex disease, and patients vary greatly and unpredictably in terms of disease subtype, activity, and progression. Pharmacologic agents that prevent disease progression and improve survival are lacking, in part because specific agents are unlikely to benefit such a heterogeneous group of patients [4]. An attractive notion is that biomarkers may provide insight into this heterogeneity, thus allowing us to cater clinical trials and ultimately therapies to specific groups of patients and provide better prognostic information. An extensive literature on biomarkers in COPD exists [1]. However, most studies have examined the association between individual biomarkers and cross-sectional outcomes. In addition, the field has been plagued by lack of validation in replication cohorts and inconsistent biomarker platforms, leading to discrepant reports (Additional file 1: Table S1) [1]. Here, we present a comprehensive analysis of the role of biomarkers, individually and in combination, in predicting both cross-sectional and longitudinal outcomes using two large, multi-center cohorts with identical platforms. We found that individual biomarkers are more closely associated with most outcomes than clinical covariates alone. Moreover, multiple biomarkers are more highly predictive than individual biomarkers for almost all COPD outcomes. With rare exceptions, the associations, including directionality, between biomarkers and outcomes identified in the discovery cohort were validated in the replication cohort (Fig. 1). Additional file 1: Tables S5 and S6 provide an easily accessible and exhaustive resource for investigators to ascertain the association between these biomarkers and almost any clinically important COPD outcome in these two cohorts. To our knowledge, ours is the first study to demonstrate an association between multiple biomarkers and cross-sectional and longitudinal outcomes in two large, multi-center cohorts.

Overall, our findings build upon prior literature, confirming some associations but improving upon existing knowledge by demonstrating that, in most cases, a distinct combination of biomarkers is associated with outcomes. In both cohorts, each of the five biomarkers studied individually correlated with airflow limitation, consistent with previous literature [4, 8, 12, 16–18]. However, a panel of five biomarkers together was more highly predictive of airflow limitation (FEV_1_) than any individual biomarker. Similarly, while previous literature suggested that sRAGE and fibrinogen are individually associated with emphysema, [5, 16] our analysis revealed that the combination of SP-D, CRP, sRAGE, and fibrinogen was more highly correlated.

Although the relationships between biomarkers and disease subtype and severity are interesting and may provide clues into the molecular pathogenesis of the different subtypes, biomarkers will be most useful clinically if they can predict longitudinal outcomes, such as future exacerbations, decline in FEV_1_, progression of emphysema, and mortality. Such risk stratification would allow clinical trials to be catered to the patients most likely to progress as well as provide patients with a more accurate and personalized prognosis. Interestingly, in the COPDGene cohort, no biomarker or combination of biomarkers added significant value to predicting an individual’s future risk of exacerbations over clinical variables including history of prior exacerbations. This is consistent with previous studies, which found that certain biomarkers were associated with exacerbations by univariate analysis but not by multivariate analysis that included clinical predictive variables, particularly prior exacerbation history [3, 17, 19]. (Although ECLIPSE was used here as a validation cohort, it is interesting to note that biomarkers were predicitve of future exacerbations, and this is likely due to differences in the subjects, such as in race and severity of disease.) One limitation of our study is the lack of blood leukocyte values, which may predict exacerbations [3, 10, 26]. Still, taken together, our findings and the literature suggest that a history of previous exacerbations is so strongly associated with future exacerbations that biomarkers may not provide substantial additional information.

COPD disease progression is highly variable [4, 5]. CC16 levels have been previously associated with decline in FEV_1_ [4, 22]. Here, the combination of CC16, fibrinogen, and sRAGE best predicted decline in lung function in the COPDGene cohort, and this combination was validated in ECLIPSE, although the addition of SP-D and CRP further improved the model. Whether pro-SP-B, previously implicated in decline in FEV_1_ [33] would further improve the model should be studied. Progression of emphysema has previously been associated with individual abnormal biomarkers [5]. We found that progression of emphysema, as measured by decline in CT density, was best modeled by the combination of CC16, sRAGE, and fibrinogen in the COPDGene cohort. This model was validated by the ECLIPSE cohort, although the addition of SP-D and CRP further enhanced the model. Whether IL-6, previously associated with progression of emphysema, [5] would further improve the model should be examined.

Although previous studies revealed that multiple biomarkers predict mortality, [9, 11] ours is the first to validate such findings in an independent large, multicenter cohort. However, there were notable discrepancies between the two cohorts. In both cohorts, the combination of SP-D, CRP, and fibrinogen improved the model over covariates and individual biomarkers. However, the best combination in COPDGene, all five biomarkers together, did not reach statistical significance in ECLIPSE. In addition, while fibrinogen or CRP alone were significantly associated with mortality in ECLIPSE (Table 6) and other [9, 13, 15] cohorts, fibrinogen and CRP were not individually predictive of mortality in COPDGene, although they did improve the model when added to the other four biomarkers. These discrepancies may be due to differences between the cohorts, such as in race and severity of disease. The concordance between the two cohorts may become stronger with ongoing follow-up, as the overall mortality rates (9.4% in COPDGene, 8.5% in ECLIPSE) are low, and 3–5 years is a relatively short duration of follow-up considering the natural history of the disease. Future studies should examine mortality over a longer period of follow-up, the contribution of additional biomarkers such as IL-6 and leukocyte count [11] to the model, and disease-specific mortality. Of note, we also report the important finding that inclusion of additional clinical variables known to be associated with mortality (e.g., age) [7] yields a novel clinical model that is more highly predictive of mortality than established models such as the BODE index. In both cohorts, biomarkers strengthen the model, and a combination of biomarkers provides enhanced predictive value over individual biomarkers.

We acknowledge that the amount of variance explained by biomarkers, as determined by correlation coefficients, is relatively low. Longer duration of follow-up and inclusion of additional biomarkers or persistence of abnormal biomarkers [10] may strengthen the correlations. However, our findings are consistent with previously reported weak correlation coefficients (R^2^ < 0.3) or relative risks (<1.5) [3, 4, 11, 15, 22, 24, 33]. Therefore, the field must acknowledge that statistically significant associations between biomarkers and outcomes that can be observed in large cohorts may be largely inadequate to explain remaining variance after strong clinical covariates are included in the models. This suggests that COPD is an exceedingly heterogeneous and complex disease, the extent of which our understanding remains quite limited. Regardless, the impact of the current study lies in the demonstration that, combinations of biomarkers correlate with COPD outcomes much (*two to ten times more*) strongly than individual biomarkers.

Limitations of this study, in addition to those discussed above, include the relatively low number of nonsmokers in the COPDGene cohort and the virtual absence of Gold 1 subjects in the ECLIPSE cohort. Neither cohort was population-based. COPDGene results should be generalizable to non-Hispanic white and African American smokers. ECLIPSE results are generalizable to white COPD patients. Although the current findings are overall generalizable because of the size of the discovery and replication cohorts, extensive clinical phenotyping, and adjustment for multiple relevant covariates, these findings should be validated in a third large cohort. Future studies should elucidate the repeatability of biomarker measurements, although most are stable over time [26].

## Conclusions

In conclusion, for the first time to our knowledge, we have demonstrated, using two large, multi-center cohorts, that multiple biomarkers are much more strongly predictive than individual biomarkers of almost all important cross-sectional and longitudinal COPD outcomes. The amount of variance explained by biomarkers is lower than clinical variables. Still, we remain optimistic that biomarkers will be useful to limit clinical trials to subgroups of patients likely to benefit from a given intervention and/or serve as surrogate endpoints if they are prospectively demonstrated to correlate with clinically relevant outcomes. As the FDA and EMA have considered approving fibrinogen and RAGE individually as biomarkers for COPD, approval of a panel of multiple biomarkers should be considered.

## Additional files

Additional file 1: Table S1. Association Between Biomarkers and COPD Outcomes. **Table S2.** Statistical Models. **Table S3.** Demographics of Subjects at Baseline: COPDGene Cohort*. **Table S4.** Demographics of Subjects at Baseline: ECLIPSE Cohort*. **Table S5.** Analysis of COPDGene cohort. Grey shading indicates each model with lines for each biomarker in that model. Columns are beta coefficient in model (B), odds ratio, standard error (SE), correlation coefficient (R^2^) or pseudo R^2^ Cragg and Uhler’s (CU) or R^2^m (the marginal portion of the R^2^), Akaike Information Criteria (AIC), and number of subjects analyzed (N). The type of model is listed on top right of table. The best model highlighted in yellow. **Table S6.** Analysis of ECLIPSE cohort. Best model in ECLIPSE cohort highlighted in yellow. Grey shading indicates each model with lines for each biomarker in that model. Columns are beta coefficient in model (B), odds ratio, standard error (SE), correlation coefficient (R^2^) or pseudo R^2^ Cragg and Uhler’s (CU) or R^2^m (the marginal portion of the R^2^), Akaike Information Criteria (AIC), and number of subjects analyzed (N). The type of model is listed on top right of table. Best model in COPDGene cohort in red font. **Table S7.** Biomarkers Associated with FEV1/FVC. **Table S8.** Biomarkers Associated with (A) Total (Moderate and Severe) Exacerbations and (B) Severe Exacerbations in the Previous 12 Months. **Table S9.** Biomarkers Associated with (A) Prospective Total (Moderate and Severe) Exacerbations or (B) Prospective Severe Exacerbations. **Table S10.** Enrollment Centers. **Table S11.** Baseline Characteristics of Subjects with Biomarker Data Compared with Entire COPDGene Cohort. **Table S12.** Correlation Between Biomarkers. **Table S13.** Biomarkers Associated with Mortality. Analysis of COPDGene and ECLIPSE cohorts by C-statistic. Covariates were BODE, age, age^2^, gender, and severe exacerbations. (ZIP 485 kb)

Additional file 2: Figure S1. Distribution of Biomarkers. Biomarker levels were log transformed. **Figure S2.** Relationship Between Individual Biomarkers and FEV_1_. Beeswarm/box plot of biomarker levels in never smokers, smokers with normal lung function PRISm, and Gold Stage 1–4 COPD patients. Central box bars represent the median and end box bars represent the first and third quartiles. Analysis by linear regression. *p < 10^−5^. **Figure S3.** Relationship Between Individual Biomarkers and Emphysema. Analysis performed by ordinal logistic regression. Covariates were FEV_1_, age, smoking status, gender, race, and BMI. % Emphysema defined as % of voxels with HU < −950. *p < 0.01. (PDF 312 kb)

Additional file 3: Supplemental Methods. (DOCX 76 kb)

## Abbreviations

AIC: Akaike information criteria
COPD: Chronic obstructive pulmonary disease
CRP: C-Reactive protein
CC16: Club cell secretory protein
EMA: European medicines agency
FDA: U.S. Food and Drug Administration
FEV_1_: Forced expiratory volume in the first second
FVC: Forced vital capacity
HU: Hounsfield units
SP-D: Surfactant protein D
sRAGE: Soluble receptor for advanced glycation endproducts
