# Supplementary material for: Multiple biomarkers predict disease severity, progression and mortality in COPD
Source: Respir Res. 2017 Jun 13;18:117. doi: 10.1186/s12931-017-0597-7 (PMC5470282; doi:10.1186/s12931-017-0597-7)
Supplement: Supplementary file 1 — Association Between Biomarkers and COPD Outcomes. Table S2. Statistical Models. Table S3. Demographics of Subjects at Baseline: COPDGene Cohort*. Table S4. Demographics of Subjects at Baseline: ECLIPSE Cohort*. Table S5. Analysis of COPDGene cohort. Grey shading indicates each model with lines for each biomarker in that model. Columns are beta coefficient in model (B), odds ratio, standard error (SE), correlation coefficient (R2) or pseudo R2 Cragg and Uhler’s (CU) or R2m (the marginal portion of the R2), Akaike Information Criteria (AIC), and number of subjects analyzed (N). The type of model is listed on top right of table. The best model highlighted in yellow. Table S6. Analysis of ECLIPSE cohort. Best model in ECLIPSE cohort highlighted in yellow. Grey shading indicates each model with lines for each biomarker in that model. Columns are beta coefficient in model (B), odds ratio, standard error (SE), correlation coefficient (R2) or pseudo R2 Cragg and Uhler’s (CU) or R2m (the marginal portion of the R2), Akaike Information Criteria (AIC), and number of subjects analyzed (N). The type of model is listed on top right of table. Best model in COPDGene cohort in red font. Table S7. Biomarkers Associated with FEV1/FVC. Table S8. Biomarkers Associated with (A) Total (Moderate and Severe) Exacerbations and (B) Severe Exacerbations in the Previous 12 Months. Table S9. Biomarkers Associated with (A) Prospective Total (Moderate and Severe) Exacerbations or (B) Prospective Severe Exacerbations. Table S10. Enrollment Centers. Table S11. Baseline Characteristics of Subjects with Biomarker Data Compared with Entire COPDGene Cohort. Table S12. Correlation Between Biomarkers. Table S13. Biomarkers Associated with Mortality. Analysis of COPDGene and ECLIPSE cohorts by C-statistic. Covariates were BODE, age, age2, gender, and severe exacerbations. (ZIP 485 kb) [file 12931_2017_597_MOESM1_ESM.zip › 2.3.2017 Supplemental Tables.pdf]

Supplemental Table 1. Association Between Biomarkers and COPD Outcomes

Biomarker	Airflow Limitation	Emphysema	Exacerbations	Decline in FEV1	Progression of Emphysema	Mortality
**CC16**	+ 1,2	- 2,3	+ 4	+ 1,5	- 3	- 6
**SP-D**	+/- 1,7,8	+ 3	+/- 4,8-10	- 1	+ 3	+ 6
**sRAGE**	+ 11-13	+ 3,13,14	+/- 9,11	+ 12	+ 3,13,14	
**CRP**	+ 1,11,15-18	- 3,4	+/- 9,10,19-22	+/- 1,16,17,23,24	+ 3	+ 6,20,21,25
**Fibrinogen**	+ 1,17,26-28	+/- 3,29	+/- 4,9,10,19,21,27,30-32	+/- 1,17,27	+ 3	+ 6,21,27,28,32-34

+: Correlation likely
+/-: Correlation uncertain due to discrepant reports
-: Correlation unlikely

1. Vestbo J, Edwards LD, Scanlon PD, et al. Changes in forced expiratory volume in 1 second over time in COPD. *N Engl J Med* 2011; **365**(13): 1184-92.
2. Lomas DA, Silverman EK, Edwards LD, Miller BE, Coxson HO, Tal-Singer R. Evaluation of serum CC-16 as a biomarker for COPD in the ECLIPSE cohort. *Thorax* 2008; **63**(12): 1058-63.
3. Coxson HO, Dirksen A, Edwards LD, et al. The presence and progression of emphysema in COPD as determined by CT scanning and biomarker expression: a prospective analysis from the ECLIPSE study. *Lancet Respir Med* 2013; **1**(2): 129-36.
4. Hurst JR, Vestbo J, Anzueto A, et al. Susceptibility to exacerbation in chronic obstructive pulmonary disease. *N Engl J Med* 2010; **363**(12): 1128-38.
5. Park HY, Churg A, Wright JL, et al. Club cell protein 16 and disease progression in chronic obstructive pulmonary disease. *Am J Respir Crit Care Med* 2013; **188**(12): 1413-9.
6. Celli BR, Locantore N, Yates J, et al. Inflammatory biomarkers improve clinical prediction of mortality in chronic obstructive pulmonary disease. *Am J Respir Crit Care Med* 2012; **185**(10): 1065-72.
7. Winkler C, Atochina-Vasserman EN, Holz O, et al. Comprehensive characterisation of pulmonary and serum surfactant protein D in COPD. *Respir Res* 2011; **12**: 29.
8. Lomas DA, Silverman EK, Edwards LD, et al. Serum surfactant protein D is steroid sensitive and associated with exacerbations of COPD. *Eur Respir J* 2009; **34**(1): 95-102.
9. Keene JD, Jacobson S, Kechris K, et al. Biomarkers Predictive of Exacerbations in the SPIROMICS and COPDGene Cohorts. *Am J Respir Crit Care Med* 2016.
10. Dickens JA, Miller BE, Edwards LD, et al. COPD association and repeatability of blood biomarkers in the ECLIPSE cohort. *Respir Res* 2011; **12**: 146.
11. Smith DJ, Yerkovich ST, Towers MA, Carroll ML, Thomas R, Upham JW. Reduced soluble receptor for advanced glycation end-products in COPD. *Eur Respir J* 2011; **37**(3): 516-22.
12. Iwamoto H, Gao J, Pulkkinen V, Toljamo T, Nieminen P, Mazur W. Soluble receptor for advanced glycation end-products and progression of airway disease. *BMC Pulm Med* 2014; **14**: 68.

13. Cheng DT, Kim DK, Cockayne DA, et al. Systemic soluble receptor for advanced glycation endproducts is a biomarker of emphysema and associated with AGER genetic variants in patients with chronic obstructive pulmonary disease. *Am J Respir Crit Care Med* 2013; **188**(8): 948-57.
14. Carolan BJ, Hughes G, Morrow J, et al. The association of plasma biomarkers with computed tomography-assessed emphysema phenotypes. *Respir Res* 2014; **15**: 127.
15. de Torres JP, Cordoba-Lanus E, Lopez-Aguilar C, et al. C-reactive protein levels and clinically important predictive outcomes in stable COPD patients. *Eur Respir J* 2006; **27**(5): 902-7.
16. Fogarty AW, Jones S, Britton JR, Lewis SA, McKeever TM. Systemic inflammation and decline in lung function in a general population: a prospective study. *Thorax* 2007; **62**(6): 515-20.
17. Jiang R, Burke GL, Enright PL, et al. Inflammatory markers and longitudinal lung function decline in the elderly. *Am J Epidemiol* 2008; **168**(6): 602-10.
18. Thorleifsson SJ, Margretardottir OB, Gudmundsson G, et al. Chronic airflow obstruction and markers of systemic inflammation: results from the BOLD study in Iceland. *Respir Med* 2009; **103**(10): 1548-53.
19. Thomsen M, Ingebrigtsen TS, Marott JL, et al. Inflammatory biomarkers and exacerbations in chronic obstructive pulmonary disease. *JAMA* 2013; **309**(22): 2353-61.
20. Dahl M, Vestbo J, Lange P, Bojesen SE, Tybjaerg-Hansen A, Nordestgaard BG. C-reactive protein as a predictor of prognosis in chronic obstructive pulmonary disease. *Am J Respir Crit Care Med* 2007; **175**(3): 250-5.
21. Agusti A, Edwards LD, Rennard SI, et al. Persistent systemic inflammation is associated with poor clinical outcomes in COPD: a novel phenotype. *PLoS One* 2012; **7**(5): e37483.
22. Eagan TM, Ueland T, Wagner PD, et al. Systemic inflammatory markers in COPD: results from the Bergen COPD Cohort Study. *Eur Respir J* 2010; **35**(3): 540-8.
23. Ahmadi-Abhari S, Kaptoge S, Luben RN, Wareham NJ, Khaw KT. Longitudinal association of C-reactive protein and lung function over 13 years: The EPIC-Norfolk study. *Am J Epidemiol* 2014;**179**(1): 48-56.
24. Higashimoto Y, Iwata T, Okada M, Satoh H, Fukuda K, Tohda Y. Serum biomarkers as predictors of lung function decline in chronic obstructive pulmonary disease. *Respir Med* 2009; **103**(8): 1231-8.
25. Man SF, Connett JE, Anthonisen NR, Wise RA, Tashkin DP, Sin DD. C-reactive protein and mortality in mild to moderate chronic obstructive pulmonary disease. *Thorax* 2006; **61**(10): 849-53.
26. Dahl M, Tybjaerg-Hansen A, Vestbo J, Lange P, Nordestgaard BG. Elevated plasma fibrinogen associated with reduced pulmonary function and increased risk of chronic obstructive pulmonary disease. *Am J Respir Crit Care Med* 2001; **164**(6): 1008-11.
27. Duvoix A, Dickens J, Haq I, et al. Blood fibrinogen as a biomarker of chronic obstructive pulmonary disease. *Thorax* 2013; **68**(7): 670-6.
28. Mannino DM, Valvi D, Mullerova H, Tal-Singer R. Fibrinogen, COPD and mortality in a nationally representative U.S. cohort. *COPD* 2012; **9**(4): 359-66.
29. Papaioannou AI, Mazioti A, Kiropoulos T, et al. Systemic and airway inflammation and the presence of emphysema in patients with COPD. *Respir Med* 2010; **104**(2): 275-82.
30. Wedzicha JA, Seemungal TA, MacCallum PK, et al. Acute exacerbations of chronic obstructive pulmonary disease are accompanied by elevations of plasma fibrinogen and serum IL-6 levels. *Thromb Haemost* 2000; **84**(2): 210-5.
31. Engstrom G, Segelstorm N, Ekberg-Aronsson M, Nilsson PM, Lindgarde F, Lofdahl CG. Plasma markers of inflammation and incidence of hospitalisations for COPD: results from a population-based cohort study. *Thorax* 2009; **64**(3): 211-5.
32. Mannino DM, Tal-Singer R, Lomas DA, et al. Plasma Fibrinogen as a Biomarker for Mortality and Hospitalized Exacerbations in People with COPD. *Chronic Obstr Pulm Dis (Miami)* 2015; **2**(1):23-34.
33. Danesh J, Lewington S, Thompson SG, et al. Plasma fibrinogen level and the risk of major cardiovascular diseases and nonvascular mortality: an individual participant meta-analysis. *JAMA* 2005;**294**(14): 1799-809.
34. Valvi D, Mannino DM, Mullerova H, Tal-Singer R. Fibrinogen, chronic obstructive pulmonary disease (COPD) and outcomes in two United States cohorts. *Int J Chron Obstruct Pulmon Dis* 2012; **7**: 173-82.

Supplemental Table 2. Statistical Models

Outcome	Model	Covariates
**Severity of Emphysema (% LAA ≤ -950 HU)**	Ordinal Regression	FEV_1, Age, Smoking Status, Gender, BMI, Race, Scanner
**FEV_1 (% Predicted)**	Linear Regression	Race
**FEV_1/FVC**	β-regression	Age, Gender, Asthma, Race
**Prospective Exacerbations**	Zero Inflated Negative Binomial	FEV_1, GERD, SGRQ, Prior Exacerbations, Race
**All Cause Mortality**	Cox Proportional Hazards	BODE, Age^2, Age, Gender, Severe Exacerbations
**Decline in FEV_1 (ml)**	Linear Mixed	Age, Time, Gender, Height, Smoking Status, Pack Years, Age^2, $Height^2$
**Decline in CT Density (%LAA)**	Linear Mixed	FEV_1, Age, Time, Smoking Status, Gender, BMI, Scanner
**Previous Exacerbations**	Zero Inflated Negative Binomial	FEV1, GERD, SGRQ, Gender, Race

BMI: Body Mass Index; FEV_1: Forced Expiratory Volume in 1 second; FVC: Forced Vital Capacity; SGRQ: St. George's Respiratory Questionnaire; LAA: Low Area Attenuation. FEV_1 and FVC are post-bronchodilator.

Supplemental Table 3. Demographics of Subjects at Baseline: COPDGene Cohort*

	Total	Nonsmokers	Control Smokers	PRISm	Gold 1	Gold 2	Gold 3	Gold 4
**N**	1465	40	664	145	137	278	132	62
**Age (Years)**	61.4 (9.2)	64.9 (10.5)	58.9 (9)	59.9 (9)	63.1 (9)	64.5 (8.9)	65.6 (7.9)	64 (7.7)
**Gender (Male/Female)**	48.7 / 51.3	25 / 75	47.6 / 52.4	40 / 60	55.5 / 44.5	51.1 / 48.9	57.6 / 42.4	50 / 50
**Race (White/African American)**	86.1 / 13.9	97.5 / 2.5	81.9 / 18.1	87.6 / 12.4	86.9 / 13.1	91.4 / 8.6	91.7 / 8.3	88.7 / 11.3
**Current Smokers (%)**	39.50	0	42.60	52.40	45.30	39.60	25.80	12.90
**Smoking (Pack Years)**	43.9 (25.1)	NA	36.6 (20.8)	46.3 (28.5)	44 (21.1)	52.7 (28.1)	55.2 (26.5)	53.7 (26.2)
**BMI**	28.8 (6)	28.7 (4.7)	28.6 (5.4)	32.8 (7.2)	27 (4.7)	29.5 (6.2)	27.4 (6.2)	25.2 (5.6)
**FEV_1 (% Predicted)**	80.5 (24.9)	102.2 (14.2)	98.8 (11.5)	71.3 (7.6)	91.4 (9.2)	65.2 (8.8)	40.2 (5.8)	21.9 (4.7)
**FVC (L)**	3.4 (1)	3.4 (0.9)	3.8 (0.9)	2.7 (0.6)	4.1 (1)	3.3 (0.9)	2.8 (0.8)	2.2 (0.7)
**FEV_1/FVC**	0.7 (0.2)	0.8 (0.1)	0.8 (0.1)	0.8 (0)	0.6 (0)	0.6 (0.1)	0.4 (0.1)	0.3 (0.1)
**Walk Distance (Feet)**	1518.4 (358.7)	1708.7 (340.7)	1644.1 (302.9)	1384.1 (354.6)	1599.7 (315.7)	1422.1 (343.1)	1261.9 (288.8)	948.1 (315.6)
**BODE**	1 (1.6)	0 (0.2)	0.3 (0.7)	1 (1.2)	0.5 (0.9)	1.3 (1.3)	3.9 (1.2)	5.7 (1)
**MRC Dyspnea Score**	1.1 (1.4)	0.2 (0.5)	0.5 (1)	1.5 (1.4)	0.8 (1.2)	1.4 (1.4)	2.5 (1.2)	3.2 (0.8)
**SGRQ Score**	22.1 (21.4)	4 (7.6)	12.7 (15.2)	27.6 (22.7)	17.7 (16.1)	28.9 (21.5)	43.6 (17.7)	55.4 (14.9)
**Emphysema (% LAA ≤ -910 HU)**	5 (8.5)	1.2 (2)	1.6 (2.1)	0.9 (1.2)	4.7 (5.4)	6.2 (7.8)	16 (12.5)	24.6 (13.7)
**Gas Trapping (%)**	22.3 (20.2)	6.7 (4.6)	10.5 (8.2)	8.6 (7)	17.8 (12.7)	28.1 (15.1)	46.6 (18.5)	63.1 (10.9)
**Pi10**	3.6 (0.1)	3.6 (0.1)	3.6 (0.1)	3.7 (0.1)	3.6 (0.1)	3.7 (0.1)	3.7 (0.1)	3.8 (0.1)
**Severe Exacerbations (#/year)**	0.1 (0.4)	0 (0)	0 (0.2)	0.1 (0.4)	0 (0.1)	0.1 (0.3)	0.3 (0.8)	0.7 (1)
**Moderate Exacerbations (#/year)**	0.3 (0.9)	0 (0.2)	0.1 (0.4)	0.3 (0.8)	0.3 (1)	0.5 (1)	0.8 (1.2)	1.6 (2)
**Chronic Bronchitis (%)**	16.5	0	11.3	16.6	13.1	25.5	27.3	27.4

*Values are expressed as Mean (S.D.). PRISm: Preserved Ratio Impaired Spirometry; BMI: Body Mass Index; FEV_1: Forced Expiratory Volume in 1 second; FVC: Forced Vital Capacity; MRC: Medical Research Council; SGRQ: St. George's Respiratory Questionnaire; LAA: Low Area Attenuation; Pi: Internal Airway Perimeter. FEV_1 and FVC are post-bronchodilator.

Supplemental Table 4. Demographics of Subjects at Baseline: ECLIPSE Cohort*

Variable	Total	Nonsmokers	Control Smokers	Gold 1	Gold 2	Gold 3	Gold 4
**N**	2746	235	348	2	954	911	296
**Age (Years)**	61.6 (8.3)	54.1 (9)	55.3 (9)	62.5 (17.7)	63.5 (7.2)	63.7 (7)	62.4 (7)
**Gender (Male/Female)**	61.6 / 38.4	36.6 / 63.4	55.5 / 44.5	50 / 50	60.2 / 39.8	67.8 / 32.2	74 / 26
**Race (White/African Descent)**	97.8 / 2.2	97.9 / 2.1	97.1 / 2.9	100 / 0	99 / 1	97.7 / 2.3	94.9 / 5.1
**Current Smokers (%)**	36	0	58.9	50	37.7	37.4	27.7
**Smoking (Pack Years)**	46.1 (27.2)	0 (0)	30.8 (21.6)	36.5 (7.8)	48.1 (28.5)	49.1 (25.7)	49.3 (27.2)
**BMI**	26.7 (5.5)	27.6 (5.5)	26.9 (4.6)	29.1 (11.7)	27.4 (5.7)	26.2 (5.5)	25 (5.7)
**FEV_1 (% Predicted)**	61.6 (29.9)	115 (13.8)	108.7 (12.2)	82.8 (4)	63.2 (8.4)	40.3 (5.8)	24.8 (3.7)
**FVC (L)**	3.3 (1)	4.1 (1)	4.2 (1)	3.5 (2)	3.4 (0.9)	2.9 (0.8)	2.3 (0.7)
**FEV_1/FVC**	52.2 (17.9)	81.1 (5.2)	79.2 (5.2)	66 (17)	52.8 (8.8)	40.4 (8.9)	32 (7.5)
**Walk Distance (Feet)**	1210.8 (399.2)	NA	1004.8 (29)	NA	1330.8 (366.9)	1167.4 (386.3)	948.2 (392.3)
**BODE**	3.2 (2.1)	NA	NA	NA	1.6 (1.4)	4 (1.6)	5.7 (1.6)
**MRC Dyspnea Score**	1.4 (1.1)	0.1 (0.3)	0.2 (0.5)	2 (1.4)	1.3 (1)	1.8 (1)	2.3 (1)
**SGRQ Score**	41 (22.6)	7.3 (5.8)	11.8 (11.3)	36.7 (7.4)	41.3 (18.6)	51.7 (16.5)	58.6 (14.3)
**Emphysema (% LAA ≤ -950 HU)**	14.5 (12.5)	4.1 (4.3)	2.5 (3.1)	6.6 (7.4)	12.2 (9.6)	20.2 (11.6)	28.4 (12.7)
**Severe Exacerbations (#/year)**	0.2 (0.6)	0 (0)	0 (0)	0 (0)	0.1 (0.4)	0.3 (0.7)	0.4 (0.9)
**Moderate Exacerbations (#/year)**	0.7 (1.1)	0 (0)	0 (0.1)	0.5 (0.7)	0.6 (1)	1 (1.3)	1.2 (1.4)
**Chronic Bronchitis (%)**	28.8	2.1	10.3	100	30.8	37	39.9

*Values are expressed as Mean (S.D.). BMI: Body Mass Index; FEV_1: Forced Expiratory Volume in 1 second; FVC: Forced Vital Capacity; MRC: Medical Research Council; SGRQ: St. George's Respiratory Questionnaire; LAA: Low Area Attenuation. FEV_1 and FVC are post-bronchodilator. NA: Data not available

Supplemental Table 7. Biomarkers Associated with FEV_1/FVC.

	COPDGene			ECLIPSE		
**Biomarker(s)**	**β**	**Pseudo R^2 CU**	**AIC**	**β**	**Pseudo R^2 CU**	**AIC**
**None**		0.140	-1687		0.215	-2192
**CC16**	0.665	0.057	-1778*	0.688	0.048	-2271*
**SP-D**	-0.191	0.005	-1683	-0.325	0.013	-2162
**sRAGE**	0.498	0.023	-1703*	1.032	0.090	-2156
**CRP**	-0.142	0.011	-1697*	-0.228	0.033	-2239*
**Fibrinogen**	-0.399	0.003	-1664	-1.120	0.040	-2064
**CC16, SP-D, CRP, sRAGE**		0.086	-1796*ǂ		0.158	-2267*

Analysis performed by β-regression.

CU: Cragg & Uhler's.

Covariates were age, gender, race, and asthma history.

*$p<0.05$ in a two-sided z-test for the null hypothesis that $\beta = 0$.

ǂBest model.

Supplemental Table 8. Biomarkers Associated with (A) Total (Moderate and Severe) Exacerbations and (B) Severe Exacerbations in the Previous 12 Months

A.

	COPDGene			ECLIPSE		
**Biomarker(s)**	**β**	**Pseudo R^2 CU**	**AIC**	**β**	**Pseudo R^2 CU**	**AIC**
**None**		0.32	1705		0.42	4993
**CC16**	0.16	0.00	1707	0.08	0.05	4849
**SP-D**	0.22	0.00	1706	0.26	0.05	4848*
**sRAGE**	0.25	0.01	1690	-0.02	0.12	4227
**CRP**	-0.36	0.00	1703*	0.13	0.06	4825*
**Fibrinogen**	-1.07	0.02	1683	0.56	0.13	4143
**sRAGE, CRP**		0.03	1688*ǂ		0.25	4102*ǂ

B.

	COPDGene			ECLIPSE		
**Biomarker(s)**	**β**	**Pseudo R^2 CU**	**AIC**	**β**	**Pseudo R^2 CU**	**AIC**
**None**		0.33	657		0.30	2069
**CC16**	1.29	0.01	654*	-0.20	0.02	2031
**SP-D**	1.87	0.00	657*	0.16	0.02	2033
**sRAGE**	-0.30	0.01	652	0.14	0.23	1621
**CRP**	-0.28	0.00	659	0.26	0.04	1990
**Fibrinogen**	-0.72	0.01	653	0.98	0.19	1680
**SP-D, Fibrinogen, sRAGE, CRP**		0.05	633*ǂ		0.41	1207*ǂ

Analysis performed by zero inflated negative binomial regression.

Covariates were FEV_1, history of GERD, gender, race, and SGRQ score.

*$p<0.05$ in a two-sided z-test for the null hypothesis that $\beta = 0$.

ǂBest model.

Supplemental Table 9. Biomarkers Associated with (A) Prospective Total (Moderate and Severe) Exacerbations or (B) Prospective Severe Exacerbations

A.

	COPDGene			ECLIPSE		
**Biomarker(s)**	**β**	**Pseudo R^2 CU**	**AIC**	**β**	**Pseudo R^2 CU**	**AIC**
**None**		0.260	2786		0.483	8474
**CC16**	-0.17	0.003	2784	0.12	0.066	8253
**SP-D**	0.27	0.004	2783	-0.09	0.066	8251
**sRAGE**	-0.43	0.032	2740	-0.35	0.084	7458*
**CRP**	0.096	0.004	2783	0.13	0.260	8179
**Fibrinogen**	-0.048	0.037	2736	0.72	0.306	7086
**sRAGE, Fibrinogen**		0.057	2708		0.423	6241*

B.

	COPDGene			ECLIPSE		
**Biomarker(s)**	**β**	**Pseudo R^2 CU**	**AIC**	**β**	**Pseudo R^2 CU**	**AIC**
**None**		0.24	1226		0.33	3961
**CC16**	-0.42	0.01	1228	0.08	0.04	3872
**SP-D**	-0.39	0.01	1229	0.27	0.04	3872
**sRAGE**	-0.83	0.02	1215	-0.53	0.20	3491*
**CRP**	0.09	0.01	1223	0.25	0.06	3828*
**Fibrinogen**	-0.17	0.02	1216	1.36	0.27	3237*
**sRAGE, CRP, Fibrinogen**		0.04	1204		0.44	2742*

Analysis performed by Negative Binomial Zero Inflation.

Covariates were FEV_1, history of GERD, and SGRQ.

*$p<0.05$ in a two-sided z-test for the null hypothesis that $\beta = 0$.

ǂBest model.

Supplemental Table 10. Enrollment Centers

Clinical Center	Institution Title	Protocol Number
**National Jewish Health**	National Jewish IRB	**HS-1883a**
**Brigham and Women's Hospital**	Partners Human Research Committee	**2007-P-000554/2; BWH**
**Baylor College of Medicine**	Institutional Review Board for Baylor College of Medicine and Affiliated Hospitals	**H-22209**
**Michael E. DeBakey VAMC**	Institutional Review Board for Baylor College of Medicine and Affiliated Hospitals	**H-22202**
**Columbia University Medical Center**	Columbia University Medical Center IRB	**IRB-AAAC9324**
**Duke University Medical Center**	The Duke University Health System Institutional Review Board for Clinical Investigations (DUHS IRB)	**Pro00004464**
**Johns Hopkins University**	Johns Hopkins Medicine Institutional Review Boards (JHM IRB)	**NA_00011524**
**Los Angeles Biomedical Research Institute**	The John F. Wolf, MD Human Subjects Committee of Harbor-UCLA Medical Center	**12756-01**
**Morehouse School of Medicine**	Morehouse School of Medicine Institutional Review Board	**07-1029**
**Temple University**	Temple University Office for Human Subjects Protections Institutional Review Board	**11369**
**University of Alabama at Birmingham**	The University of Alabama at Birmingham Institutional Review Board for Human Use	**FO70712014**
**University of California, San Diego**	University of California, San Diego Human Research Protections Program	**070876**
**University of Iowa**	The University of Iowa Human Subjects Office	**200710717**
**Ann Arbor VA**	VA Ann Arbor Healthcare System IRB	**PCC 2008-110732**
**University of Minnesota**	University of Minnesota Research Subjects' Protection Programs (RSPP)	**0801M24949**
**University of Pittsburgh**	University of Pittsburgh Institutional Review Board	**PRO07120059**
**University of Texas Health Sciences Center at San Antonio**	UT Health Science Center San Antonio Institutional Review Board	**HSC20070644H**
**Health Partners Research Foundation**	Health Partners Research Foundation Institutional Review Board	**07-127**
**University of Michigan**	Medical School Institutional Review Board (IRBMED)	**HUM00014973**
**Minneapolis VA Medical Center**	Minneapolis VAMC IRB	**4128-A**
**Reliant Clinic**	Institutional Review Board/Research Review Committee<br>Saint Vincent Hospital – Fallon Clinic – Fallon Community Health Plan	**1143**

Supplemental Table 11. Baseline Characteristics of Subjects with Biomarker Data Compared with Entire COPDGene Cohort

Variable	Biomarker Subset	COPDGene Cohort	p-value
**Nonsmokers**	2.7%	1.1%	1.00×10^{-9}
**Age (Years)**	61.4 (9.3)	60.0 (9.0)	3.89×10^{-14}
**Gender (% Male)**	49	53	5.79×10^{-4}
**Race (% White)**	86	67	1.67×10^{-88}
**Current Smokers (%)**	40	53	1.13×10^{-23}
**Chronic Bronchitis (%)**	17	19	0.01
**Severe Exacerbations (%)**	9	12	7.91×10^{-4}
**Smoking (Pack Years)**	43.9 (25.1)	44.2 (25.0)	NS
**BMI**	28.8 (6.0)	28.8 (6.3)	NS
**FEV_1 (% Predicted)**	80.5 (24.9)	76.6 (25.6)	5.64×10^{-9}
**FVC (% Predicted)**	90.1 (17.2)	87.1 (18.3)	5.12×10^{-11}
**FEV_1/FVC**	0.68 (0.16)	0.67 (0.16)	NS
**Walk Distance (Meters)**	1518.4 (358.7)	1353.2 (399.3)	1.89×10^{-61}
**BODE**	1.05 (1.63)	1.44 (1.85)	1.64×10^{-19}
**Dyspnea (%)**	48%	55%	1.40×10^{-08}
**SGRQ Score**	22.1 (21.4)	27.1 (23.0)	3.14×10^{-19}
**Emphysema (% LAA ≤ -950 HU)**	4.96 (8.50)	6.12 (9.57)	2.63×10^{-07}
**Gas Trapping (%)**	16.9 (18.2)	21.78 (19.9)	3.14×10^{-22}
**Pi10**	3.64 (0.13)	3.68 (0.13)	4.78×10^{-30}

*Values are expressed as Mean (S.D.). BMI: Body Mass Index; FEV_1: Forced Expiratory Volume in 1 second; FVC: Forced Vital Capacity; MRC: Medical Research Council; SGRQ: St. George's Respiratory Questionnaire; LAA: Low Area Attenuation; Pi: Internal Airway Perimeter. FEV_1 and FVC are post-bronchodilator. Severe Exacerbations (%) is the percent of subjects that have had a severe exacerbation. Dyspnea (%) is the percent of subjects that have an MRC Dyspnea Score > 0. t-test was performed for continuous variables; Chi-square was performed for ordinal variables.

Supplemental Table 12. Correlation Between Biomarkers

R^2 (Correlation)

Biomarker	CC16	SP-D	sRAGE	CRP	Fibrinogen
**CC16**	1	0.09	0.32	0.02	0.08
**SP-D**	0.09	1	0.12	-0.01	0.09
**sRAGE**	0.32	0.12	1	0.01	0.03
**CRP**	0.02	-0.01	0.01	1	0.4
**Fibrinogen**	0.08	0.09	0.03	0.4	1

n

Biomarker	CC16	SP-D	sRAGE	CRP	Fibrinogen
**CC16**	1463	1459	1446	1461	1434
**SP-D**	1459	1460	1443	1459	1433
**sRAGE**	1446	1443	1447	1445	1424
**CRP**	1461	1459	1445	1462	1434
**Fibrinogen**	1434	1433	1424	1434	1434

p-value

Biomarker	CC16	SP-D	sRAGE	CRP	Fibrinogen
**CC16**		0.0003	< 0.0001	0.4185	0.0017
**SP-D**	0.0003		2×10^{-6}	0.6640	0.0004
**sRAGE**	< 0.0001	2×10^{-6}		0.5946	0.3161
**CRP**	0.4185	0.6640	0.5946		< 0.0001
**Fibrinogen**	0.0017	0.0004	0.3161	< 0.0001	

Analysis performed by Pearson Correlations.
