# Supplementary material for: Multiple biomarkers predict disease severity, progression and mortality in COPD
Source: Respir Res. 2017 Jun 13;18:117. doi: 10.1186/s12931-017-0597-7 (PMC5470282; doi:10.1186/s12931-017-0597-7)

Supplemental Figure 1. Relationship Between Individual Biomarkers and FEV_1.

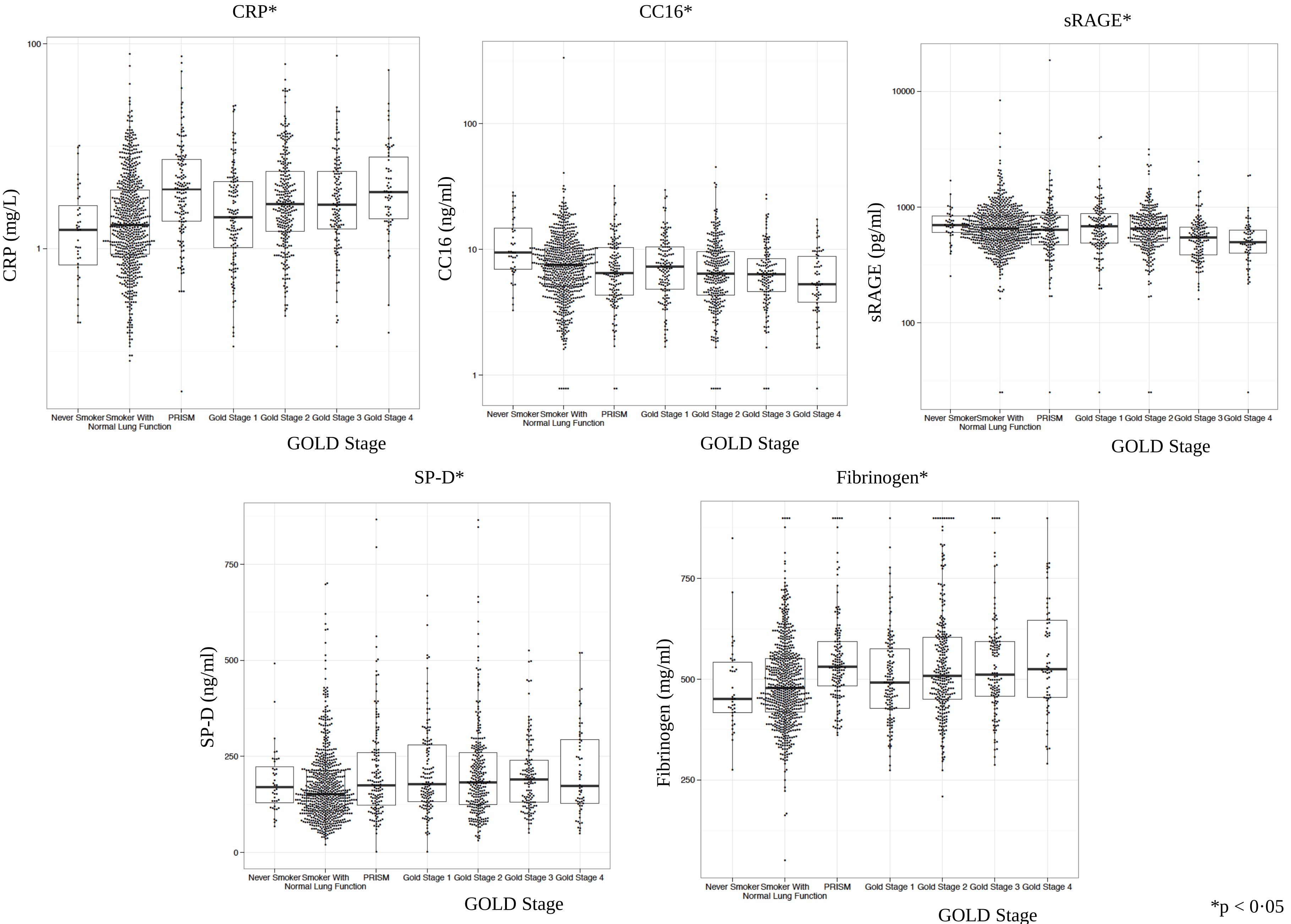

Supplemental Figure 2. Relationship Between Individual Biomarkers and Emphysema. Analysis performed by ordinal logistic regression. Covariates were FEV_1, age, smoking status, gender and BMI. % Emphysema defined as % of voxels with HU < -950. *p<0.01.

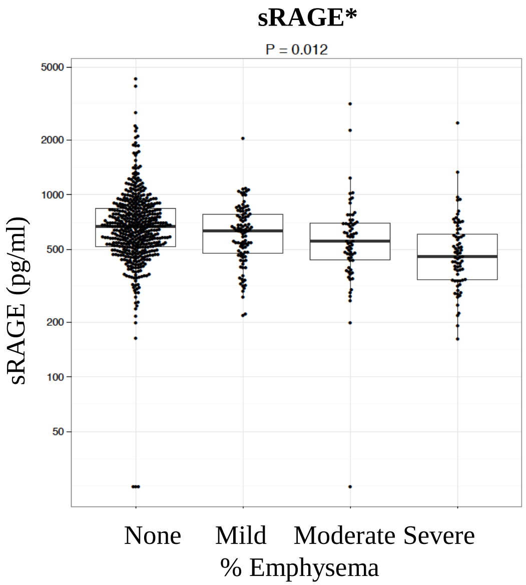

Supplemental Fig. 3. Distribution of Biomarkers: COPDGene. Biomarkers are Log Normally Distributed**.**

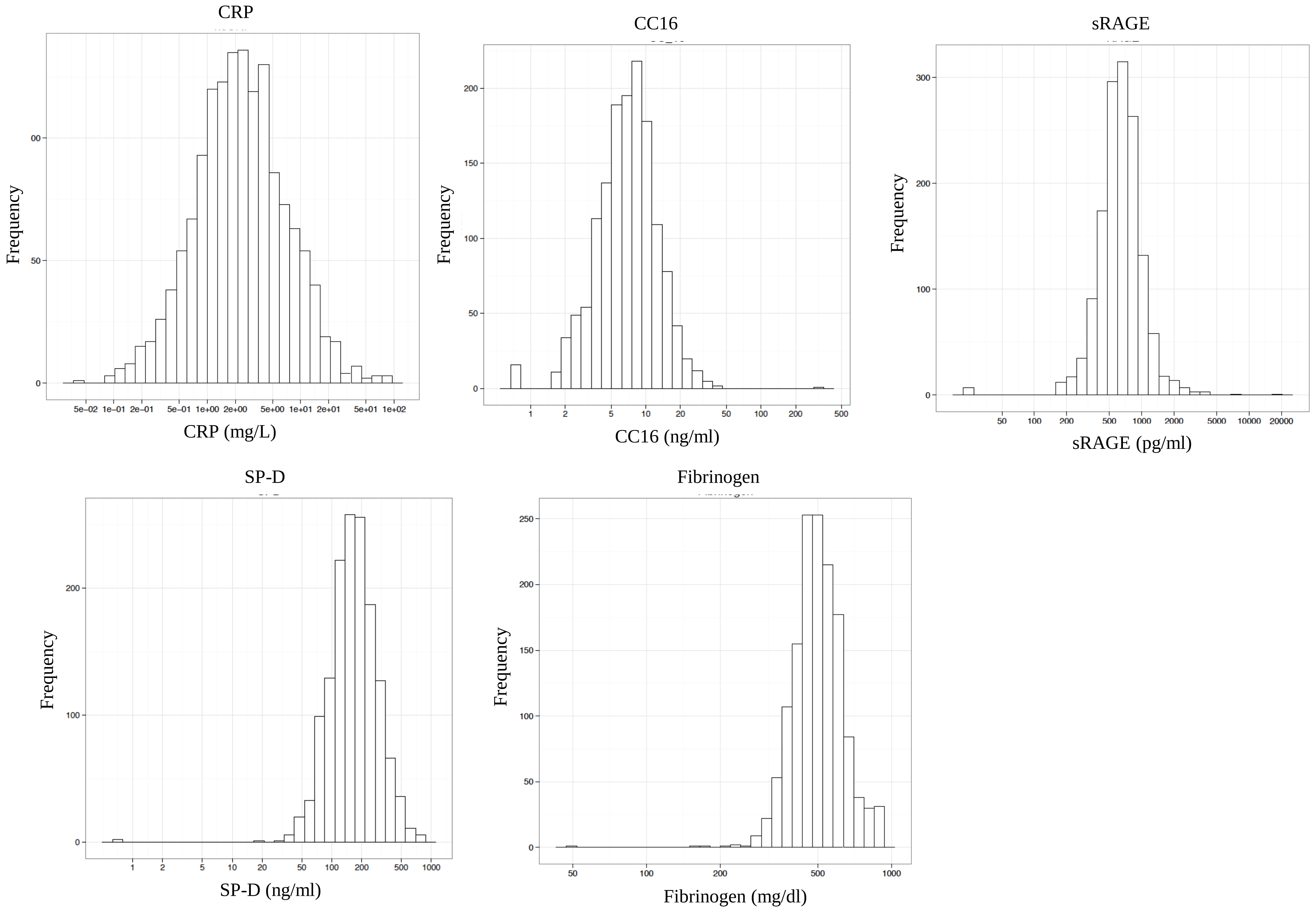

Supplement: Supplementary file 2 — Distribution of Biomarkers. Biomarker levels were log transformed. Figure S2. Relationship Between Individual Biomarkers and FEV1. Beeswarm/box plot of biomarker levels in never smokers, smokers with normal lung function PRISm, and Gold Stage 1–4 COPD patients. Central box bars represent the median and end box bars represent the first and third quartiles. Analysis by linear regression. *p < 10−5. Figure S3. Relationship Between Individual Biomarkers and Emphysema. Analysis performed by ordinal logistic regression. Covariates were FEV1, age, smoking status, gender, race, and BMI. % Emphysema defined as % of voxels with HU < −950. *p < 0.01. (PDF 312 kb) [file 12931_2017_597_MOESM2_ESM.pdf]
