# Supplementary material for: Multiple biomarkers predict disease severity, progression and mortality in COPD
Source: Respir Res. 2017 Jun 13;18:117. doi: 10.1186/s12931-017-0597-7 (PMC5470282; doi:10.1186/s12931-017-0597-7)
Supplement: Supplementary file 3 — Supplemental Methods. (DOCX 76 kb) [file 12931_2017_597_MOESM3_ESM.docx]

**Additional file 3: Supplemental Methods**

**Study Population and Phenotypes:** COPDGene (NCT02445183) enrolled 10,300 non-Hispanic white and African-American subjects ages 45-80 with a ≥ 10 pack year smoking history as well as nonsmokers.[1] Subjects were enrolled from January 2008 to April 2011. Nonsmokers were defined as subjects who had smoked cigarettes <100 cigarettes in a lifetime. COPD was defined as a post-bronchodilator FEV_1_/FVC ratio < 0.70. Smoker controls were current or former smokers without evidence of airflow limitation (FEV_1_/FVC ≥ 0.70). Smokers with Preserved FEV_1_/FVC ratio but Impaired Spirometry (i.e., FEV_1_ < 80% predicted with FEV_1_/FVC > 0.70) were classified as “PRISm”.[2] Exclusion criteria included pregnancy, history of other lung disease except asthma, exacerbation in previous 30 days, previous resection of at least one lobe of lung, active cancer, suspected lung cancer, metal in the chest, recent eye surgery, MI, or other cardiac hospitalization, recent chest or abdominal surgery, inability to use albuterol, multiple self-described racial categories, history of chest radiation therapy, and first or second relative already enrolled in the study.[1] Subjects provided informed consent, and institutional review boards of participating sites approved the study [Supplemental Table 10 and [3]]. Questionnaires included symptoms, comorbidities, history of exacerbations, and a St. George’s Respiratory Questionnaire and Modified Medical Research Questionnaire.[1] Height, weight, blood pressure, and oxygen saturation were assessed. Pre- and post-bronchodilator spirometry, inspiratory and expiratory high resolution CT scans, six minute walk test, and vital signs were performed, and a BMI was calculated. CT scans were acquired on multi-detector scanners with at least 16 channels. Volumetric acquisitions were obtained at full inspiration (200 mAs) and at the end of expiration (50 mAs). Whole blood was collected from 1,465 subjects by venipuncture into p100 vacutainer tubes containing ethylenediaminetetraacetic acid, centrifuged at 2000 x g for 15 minutes. Plasma was stored at -80°C until analyzed. Samples from both cohorts were analyzed for biomarkers on the same platform in the same laboratory with identical quality controls. sRAGE, SP-D, high sensitivity (hs) CRP, fibrinogen, and CC16 levels were measured. The biomarker immunoassay performance information was previously published.[3] Values that fell below the lower limit of detection of the assay were assigned a value of one-half of the lower limit of detection. Repeatability of the assay, as determined by a value at 3 months that falls within 25% of the initial value, has been documented.[4] The baseline characteristics of the subjects with biomarker data is shown in Supplemental Table 11. Specific methods for spirometry and CT scanning and quality control have been previously published.[1, 5] The BODE index was calculated.[6] Severity of COPD was determined by the Global Initiative for Chronic Obstructive Lung Disease (GOLD) criteria.[7, 8] VIDA software (VIDA Diagnostics) was used to measure lung attenuation, relative lung volumes falling below -950 or -910 HU, and airway wall thickness. Emphysema was defined by the percent of voxels with Hounsfield Units (HU) < -950 or percent low attenuation area (%LAA), on CT.[9] Severity of emphysema was classified as none (LAA < 5%), mild (LAA 5-10%), moderate (LAA 10-20%), or severe (LAA > 20%) based on previous studies [10, 11]. Air trapping was measured by 3D Slicer. Air trapping was defined by the percent of voxels with HU < -856 on expiratory images. Airway wall thickness at an internal perimeter of 10 mm (pi10) was calculated as described previously.[12] Subjects were classified as having chronic bronchitis if they reported cough productive of sputum present daily for at least 3 months per year, at least 2 years in a row. Longitudinal follow-up (LFU) interviews by telephone or internet were conducted every six months. The number of exacerbations per year was determined. Moderate exacerbations were defined as those treated with steroids and/or antibiotics; severe exacerbations were defined as those resulting in hospitalization. LFU in-person visits for spirometry and CT scanning were conducted every five years. Decline in FEV_1_ (ml/year) was calculated. Progression of emphysema was calculated as change in %LAA/year. All-cause mortality was determined. The median follow-up period was 3.3 years for exacerbations, 5.08 years for spirometry and CT scanning, and 4.92 years for mortality. ECLIPSE (NCT00292552) enrolled 2,746 subjects, all of whom had biomarker data, from 2005-2010. Details of the ECLIPSE study have been previously described.[13] For the ECLIPSE cohort, the median follow-up period was 3.0 years for exacerbations, spirometry, CT scanning, and mortality. Covariates were chosen based on associations documented in the literature [3, 14-23]**.**  Our study was conducted according to the Strengthening the Reporting of Observational Studies in Epidemiology (STROBE) statement.[24]

In the COPDGene cohort of 1465 subjects that had biomarkers, 1458 were analyzed for FEV_1_/FVC ratio, 1456 were analyzed for FEV_1_, 1420 were analyzed for emphysema, 1458 were analyzed for previous exacerbations, 1330 were analyzed for prospective exacerbations, 1238 were analyzed for decline in FEV_1_, 1223 were analyzed for progression of emphysema, and 1420 were analyzed for mortality (Supplemental Table 5). Subjects were followed from 3.67 to 7.67 years. There were a total of 4,177 subject-years followed. Loss to follow-up was minimized by searching voting and credit card records. In the case of missing data, subjects were excluded from analysis. The numbers of subjects analyzed for each outcome in the ECLIPSE cohort are listed in Supplemental Table 6.

**Modeling and Analysis:** R (v 3.2.0) was used. p-values were determined by two-sided t-tests (or z-tests for the beta, negative binomial and logistic regression, and Cox proportional hazards) for the null hypothesis that β coefficients for biomarker-outcome associations were zero. Because of non-normality, biomarker values were log transformed (Supplemental Figure 3). Correlation between biomarkers was assessed by Pearson’s correlation. Differences in demographic characteristics of study subjects were analyzed using a t-test for continuous variables and a Chi squared test for categorical variables. There were significant correlations (p-value <0.001 and correlation >0.10) between (1) CC16 and sRAGE and (2) SP-D and sRAGE and (3) CRP and fibrinogen (Supplemental Table 12).

FEV_1_ percent predicted was modeled by linear regression, FEV_1_/FVC was modeled by β-regression, severity of emphysema was modeled by ordinal regression, and prospective and prior exacerbations were modeled by zero inflated negative binomial regression, with follow-up time used as an offset in prospective exacerbations. Linear mixed models were used for decline in FEV_1_ and decline in CT density. See Supplemental Table 2 for models and covariates used. Covariates for each model were chosen based on previous literature,[3, 6, 14, 16, 25-27] with covariates that were significant in more than one study applied. Individuals with missing covariates, biomarkers, or outcomes were excluded. We did not adjust for multiple hypothesis testing because there were only five biomarkers, and the combinations were analyzed by nested models that are not independent.

For linear regression, the standard R^2^ was used to report the percent variance explained by a biomarker, or combination of biomarkers, over clinical covariates alone. For the ordinal, negative binomial, and β-regression models, pseudo R^2^ Cragg and Uhler’s (CU) was determined instead. For the linear mixed models, R^2^m, the marginal portion of the R^2^, was calculated.

Cox proportional hazards ratio was calculated to determine the correlation between age, age^2^, gender, and history of exacerbations and mortality after adjusting for BODE index. Cox proportional hazards ratio was calculated to determine the correlation between biomarker concentration and mortality after adjusting for age, age^2^, gender, BODE, and history of exacerbations.

In some cases, the best model in the COPDGene cohort was validated by ECLIPSE to statistically significantly improve predictive value, but additional or different combinations of biomarkers were found to enhance prediction in ECLIPSE. All analyses performed on the COPDGene and ECLIPSE cohorts are shown in Supplemental Tables 5,6, respectively, with the best model in each cohort highlighted in yellow. The best model in COPDGene is shown in red font on the ECLIPSE analysis (Supplemental Table 6). Supplemental Tables 5 and 6 provide an easily accessible and exhaustive resource for investigators to ascertain the association between these biomarkers with almost any clinically important COPD outcome in these two cohorts.

**REFERENCES**

1. Regan EA, Hokanson JE, Murphy JR, Make B, Lynch DA, Beaty TH, Curran-Everett D, Silverman EK, Crapo JD: **Genetic epidemiology of COPD (COPDGene) study design.** *COPD* 2010, **7:**32-43.

2. Wan ES, Castaldi PJ, Cho MH, Hokanson JE, Regan EA, Make BJ, Beaty TH, Han MK, Curtis JL, Curran-Everett D, et al: **Epidemiology, genetics, and subtyping of preserved ratio impaired spirometry (PRISm) in COPDGene.** *Respir Res* 2014, **15:**89.

3. Celli BR, Locantore N, Yates J, Tal-Singer R, Miller BE, Bakke P, Calverley P, Coxson H, Crim C, Edwards LD, et al: **Inflammatory biomarkers improve clinical prediction of mortality in chronic obstructive pulmonary disease.** *Am J Respir Crit Care Med* 2012, **185:**1065-1072.

4. Dickens JA, Miller BE, Edwards LD, Silverman EK, Lomas DA, Tal-Singer R, Evaluation of CLtISEsi: **COPD association and repeatability of blood biomarkers in the ECLIPSE cohort.** *Respir Res* 2011, **12:**146.

5. Lomas DA, Silverman EK, Edwards LD, Locantore NW, Miller BE, Horstman DH, Tal-Singer R, Evaluation of CLtIPSEsi: **Serum surfactant protein D is steroid sensitive and associated with exacerbations of COPD.** *Eur Respir J* 2009, **34:**95-102.

6. Celli BR, Cote CG, Marin JM, Casanova C, Montes de Oca M, Mendez RA, Pinto Plata V, Cabral HJ: **The body-mass index, airflow obstruction, dyspnea, and exercise capacity index in chronic obstructive pulmonary disease.** *N Engl J Med* 2004, **350:**1005-1012.

7. Pauwels RA, Buist AS, Ma P, Jenkins CR, Hurd SS, Committee GS: **Global strategy for the diagnosis, management, and prevention of chronic obstructive pulmonary disease: National Heart, Lung, and Blood Institute and World Health Organization Global Initiative for Chronic Obstructive Lung Disease (GOLD): executive summary.** *Respir Care* 2001, **46:**798-825.

8. Fabbri LM, Hurd SS, Committee GS: **Global Strategy for the Diagnosis, Management and Prevention of COPD: 2003 update.** *Eur Respir J* 2003, **22:**1-2.

9. Madani A, Zanen J, de Maertelaer V, Gevenois PA: **Pulmonary emphysema: objective quantification at multi-detector row CT--comparison with macroscopic and microscopic morphometry.** *Radiology* 2006, **238:**1036-1043.

10. Schroeder JD, McKenzie AS, Zach JA, Wilson CG, Curran-Everett D, Stinson DS, Newell JD, Jr., Lynch DA: **Relationships between airflow obstruction and quantitative CT measurements of emphysema, air trapping, and airways in subjects with and without chronic obstructive pulmonary disease.** *AJR Am J Roentgenol* 2013, **201:**W460-470.

11. Carolan BJ, Hughes G, Morrow J, Hersh CP, O'Neal WK, Rennard S, Pillai SG, Belloni P, Cockayne DA, Comellas AP, et al: **The association of plasma biomarkers with computed tomography-assessed emphysema phenotypes.** *Respir Res* 2014, **15:**127.

12. Nakano Y, Wong JC, de Jong PA, Buzatu L, Nagao T, Coxson HO, Elliott WM, Hogg JC, Pare PD: **The prediction of small airway dimensions using computed tomography.** *Am J Respir Crit Care Med* 2005, **171:**142-146.

13. Vestbo J, Anderson W, Coxson HO, Crim C, Dawber F, Edwards L, Hagan G, Knobil K, Lomas DA, MacNee W, et al: **Evaluation of COPD Longitudinally to Identify Predictive Surrogate End-points (ECLIPSE).** *Eur Respir J* 2008, **31:**869-873.

14. Coxson HO, Dirksen A, Edwards LD, Yates JC, Agusti A, Bakke P, Calverley PM, Celli B, Crim C, Duvoix A, et al: **The presence and progression of emphysema in COPD as determined by CT scanning and biomarker expression: a prospective analysis from the ECLIPSE study.** *Lancet Respir Med* 2013, **1:**129-136.

15. Vestbo J, Agusti A, Wouters EF, Bakke P, Calverley PM, Celli B, Coxson H, Crim C, Edwards LD, Locantore N, et al: **Should we view chronic obstructive pulmonary disease differently after ECLIPSE? A clinical perspective from the study team.** *Am J Respir Crit Care Med* 2014, **189:**1022-1030.

16. Hurst JR, Vestbo J, Anzueto A, Locantore N, Mullerova H, Tal-Singer R, Miller B, Lomas DA, Agusti A, Macnee W, et al: **Susceptibility to exacerbation in chronic obstructive pulmonary disease.** *N Engl J Med* 2010, **363:**1128-1138.

17. Bowler RP, Kim V, Regan E, Williams AA, Santorico SA, Make BJ, Lynch DA, Hokanson JE, Washko GR, Bercz P, et al: **Prediction of acute respiratory disease in current and former smokers with and without COPD.** *Chest* 2014, **146:**941-950.

18. Kesteloot H, Huang X: **On the relationship between human all-cause mortality and age.** *Eur J Epidemiol* 2003, **18:**503-511.

19. Wang H, Dwyer-Lindgren L, Lofgren KT, Rajaratnam JK, Marcus JR, Levin-Rector A, Levitz CE, Lopez AD, Murray CJ: **Age-specific and sex-specific mortality in 187 countries, 1970-2010: a systematic analysis for the Global Burden of Disease Study 2010.** *Lancet* 2012, **380:**2071-2094.

20. Camilli AE, Burrows B, Knudson RJ, Lyle SK, Lebowitz MD: **Longitudinal changes in forced expiratory volume in one second in adults. Effects of smoking and smoking cessation.** *Am Rev Respir Dis* 1987, **135:**794-799.

21. Kerstjens HA, Rijcken B, Schouten JP, Postma DS: **Decline of FEV1 by age and smoking status: facts, figures, and fallacies.** *Thorax* 1997, **52:**820-827.

22. Burrows B, Lebowitz MD, Camilli AE, Knudson RJ: **Longitudinal changes in forced expiratory volume in one second in adults. Methodologic considerations and findings in healthy nonsmokers.** *Am Rev Respir Dis* 1986, **133:**974-980.

23. Einvik G, Kongerud J, Bakke B, Soyseth V: **The influence by body mass index on annual lung function decline is not related to smoking status.** *Eur Respir J* 2016, **48:**PA4233.

24. von Elm E, Altman DG, Egger M, Pocock SJ, Gotzsche PC, Vandenbroucke JP, Initiative S: **The Strengthening the Reporting of Observational Studies in Epidemiology (STROBE) statement: guidelines for reporting observational studies.** *Lancet* 2007, **370:**1453-1457.

25. Vestbo J, Edwards LD, Scanlon PD, Yates JC, Agusti A, Bakke P, Calverley PM, Celli B, Coxson HO, Crim C, et al: **Changes in forced expiratory volume in 1 second over time in COPD.** *N Engl J Med* 2011, **365:**1184-1192.

26. Agusti A, Edwards LD, Rennard SI, MacNee W, Tal-Singer R, Miller BE, Vestbo J, Lomas DA, Calverley PM, Wouters E, et al: **Persistent systemic inflammation is associated with poor clinical outcomes in COPD: a novel phenotype.** *PLoS One* 2012, **7:**e37483.

27. Puhan MA, Garcia-Aymerich J, Frey M, ter Riet G, Anto JM, Agusti AG, Gomez FP, Rodriguez-Roisin R, Moons KG, Kessels AG, Held U: **Expansion of the prognostic assessment of patients with chronic obstructive pulmonary disease: the updated BODE index and the ADO index.** *Lancet* 2009, **374:**704-711.
